# Biomechanical Analysis and Solution Suggestions of Screw Replacement Scenarios in Femoral Neck Fracture Surgeries: Finite Element Method

**DOI:** 10.1111/os.14337

**Published:** 2025-01-06

**Authors:** Yılmaz Güvercin, Murat Yaylacı, Ayberk Dizdar, Mehmet Emin Özdemir, Sevil Ay, Ecren Uzun Yaylacı, Umitcan Karahasanoğlu, Hüseyin Uygun, Gökhan Peker

**Affiliations:** ^1^ Department of Orthopaedic and Traumatology Trabzon Kanuni Training and Research Hospital Trabzon Turkey; ^2^ Biomedical Engineering MSc Program Recep Tayyip Erdogan University Rize Turkey; ^3^ Department of Civil Engineering Recep Tayyip Erdogan University Rize Turkey; ^4^ Turgut Kıran Maritime Faculty Recep Tayyip Erdogan University Rize Turkey; ^5^ Department of Biomedical Engineering Kocaeli University Kocaeli Turkey; ^6^ Department of Civil Engineering Cankiri Karatekin University Çankırı Turkey; ^7^ Department of Civil Engineering Artvin Coruh University Artvin Turkey; ^8^ Faculty of Fisheries Recep Tayyip Erdogan University Rize Turkey

**Keywords:** biomechanics, femoral neck fracture, finite element method, medial buttress plates, screw

## Abstract

**Objective:**

Despite several surgical options, there has yet to be a consensus on the best treatment for femoral neck fracture (FNF) due to higher complication rates compared to other bone fractures. This study aims to examine the possible consequences and solution suggestions of changing screws during surgery for various reasons in FNF surgical treatment from a biomechanical perspective.

**Method:**

FNF and treatment materials were analyzed biomechanically using a package program based on the finite element method (FEM). This study created a solid model with images of femur bone tomography. Dynamic hip screws (DHS), cannulated screws (CCS), and medial buttress plates (MBP) were obtained by making three‐dimensional designs. The required elements for the models were assigned, and the material properties of the elements were defined. The solutions were obtained as crack distance and deformation results after defining the boundary conditions and applying the necessary loading.

**Results:**

The strain and crack distance values created by six models on the fracture line under different parameters were obtained, and the numerical results were evaluated. The DHS and CCS models produced the highest crack distance and deformation values when all screws were loose. The lowest values were obtained in the intact‐85 model when all CCS were tight. When the results are evaluated, it is seen that the MBP has a decreasing effect on the results. Mechanical evaluation of six different options used in femoral neck fractures was performed. 85 mm CCS applied to our standard model gave the best results, while the use of 80 mm CCS in the same model showed promising results compared to other models. It is understood that CCS have the best stability even in loosening models with the medial support plate. Different models are from intact‐85 mm DHS+1CS+MBP to DHS+1CS, which was worked with LSR+USR‐2, according to decreasing stability.

**Conclusion:**

This study offers various biomechanical solutions to possible intraoperative problems in FNF treatment. The following results were obtained from the study data. When the CCS needs to be lengthened or replaced, it is appropriate to use the CCS with the MBP. A single anti‐rotation screw is sufficient for lag screw extensions of the DHS plate, and the MBP may be a savior procedure in surgery.

## Graphical abstract

Despite several surgical options, there has yet to be a consensus on the best treatment for femoral neck fracture (FNF) due to higher complication rates compared to other bone fractures. This study aims to examine the possible consequences and solution suggestions of changing screws during surgery for various reasons in FNF surgical treatment from a biomechanical perspective. This study offers various biomechanical solutions to possible intraoperative problems in FNF treatment. The following results were obtained from the data obtained from the study. When the CCS needs to be lengthened or replaced, it is appropriate to use the CCS with the MBP. A single anti‐rotation screw is sufficient for lag screw extensions of the DHS plate, and the MBP may be a savior procedure in surgery.

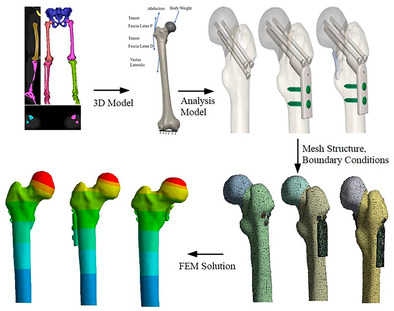

## 1 Introduction

Femoral neck fractures (FNF) are bone injuries between the femoral head and the trochanteric region. It is a common injury among elderly individuals, which poses significant socioeconomic challenges for young people. The number of injuries is predicted to increase exponentially in the coming years [1]. It tends to be mostly displaced in young adults because it occurs with high‐energy trauma mechanisms. Treatment of young adult FNF is controversial because the complication rate is higher than other fractures. For example, in 20%–60% of cases, avascular necrosis, mal‐union, and non‐union can be observed [2, 3]. Different implant systems are used in the treatment of FNF. Surgeons prefer percutaneous cannulated screws (CCS) as they provide strong fixation, are minimally invasive, have a short operating time, require relatively less scope, and cause less bleeding [4]. Many alternative systems to CCS have entered the literature in recent years. In orthopedic surgery, dynamic hip screws (DHS) are preferred over CCS for displaced fractures [5]. In a comparison of CCS and DHS in a large case series, the DHS group exhibited a higher incidence of avascular necrosis [6]. An article comparing partially threaded CCS with headless compression screws reported that headless compression screws could reduce the complication rate. In contrast, another article reported that CCS shortened the union time [7, 8]. In a comparison study between CCS‐applied femoral neck systems, it was reported that the complications of fracture non‐union, femoral head avascular necrosis, and screw loosening were the same in the two groups [9]. Medial buttress plate (MBP) is mainly used to increase the stability of CS and can reduce complication rates, as reported in the literature [10, 11].

After applying CS, there is a possibility of experiencing loss of fixation and related complications [12]. Loss of fixation occurs for various reasons. Examples of these reasons include posterior pelvic tilt, implant features, screw thickness, and length, and placement on the femoral neck [13, 14]. During surgery, if the screws move in different directions while drilling or being placed, or if there is loss of reduction or the screw length is not suitable after compression, replacement of the screw is necessary, even if it is not the preferred option.

Finite element method (FEM) studies are a powerful tool for investigating musculoskeletal disorders. FEM predicts fracture risk, union, and implant properties accurately [15, 16, 17, 18, 19, 20]. Nowadays FEM, which is used for the solution and analysis of many problems in different engineering fields, especially in rigid body mechanics; It is also widely used in biomechanical studies [21, 22, 23, 24]. Biomechanical studies can be classified as sports, occupational, and clinical biomechanics. One of the prominent subjects in clinical biomechanical studies is the musculoskeletal system [25, 26, 27, 28, 29].

No articles reporting the impact of screw replacement during surgery on complications were found in the literature. The purpose of the study: (i) Different intraoperative simulations of the most used surgical methods in FNF were analyzed using the FEM method; (ii) The effects of replacing screws during surgery on complications and solution methods were mechanically investigated.

## 2 Materials and Methods

The reasons why those working on musculoskeletal systems prefer analysis with FEM are not only time and cost savings but also because it gives results very close to experimental or analytical analyses, and realistic finite element models can manifest. With this, finite element analysis (FEA) can lead to technological developments by enabling new products in orthopedic and surgical fields [30, 31, 32, 33].

The femur bone is the most important bone that provides our balance and supports our movement in different postures such as sitting, walking, and standing. In addition, the bone carries the highest percentage of our body weight and is, therefore, subject to maximum deformation and stress in the body. In this study, different screw applications investigated used FEA of the fracture seen in the femur bone, which is the longest and strongest bone in our musculoskeletal system and was discussed biomechanically.

### 2.1 Three‐Dimensional (3D) Model Creation

**FIGURE 1:**
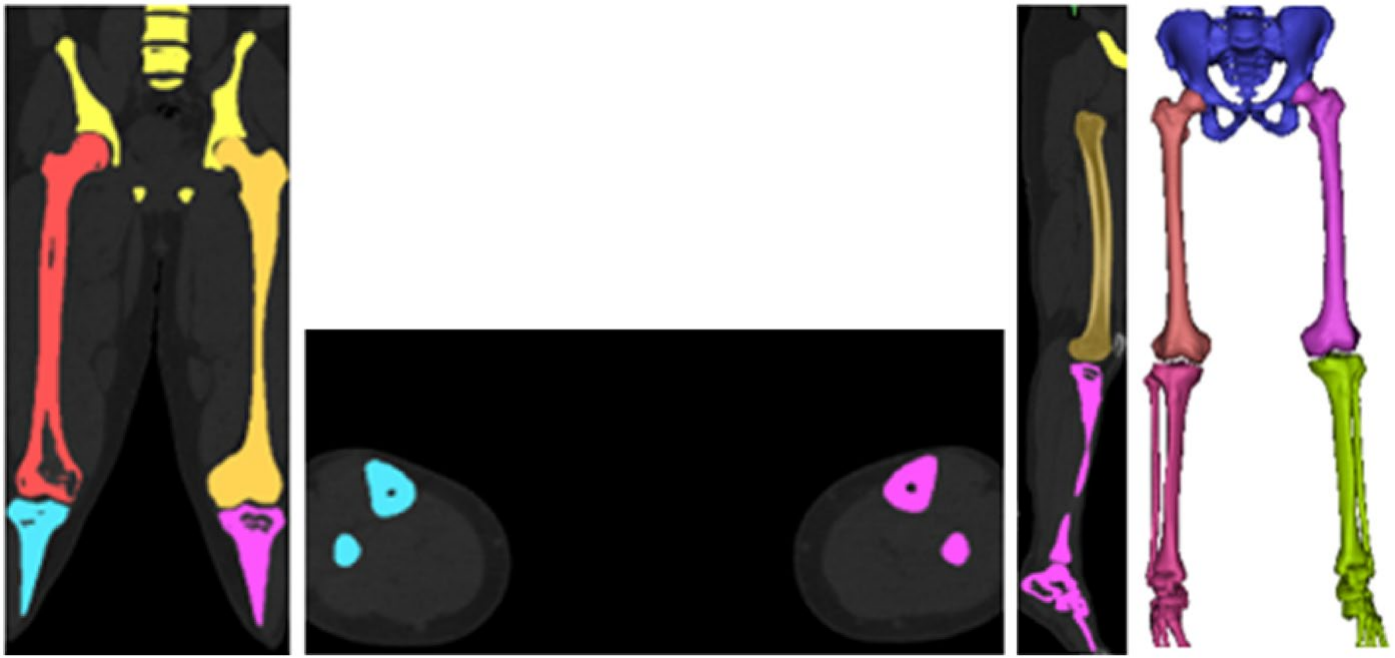
3D model.

The FNF at the proximal end of a male (178 cm, 80 kg, 30 years) patient using magnetic resonance imaging (MRI) scan data was evaluated as Pauwells type III fracture according to the Pauwells classification. The approval of the institution (Recep Tayyip Erdoğan Training and Research Hospital) where the radiograph was taken, and the patient's consent was obtained. According to the scan data, there is a 70° Pauwels angle between the centerline of the femoral shaft and the fracture. The CT scan data of the proximal end of the femur were taken in DICOM format and transferred to Mimics Innovation Suite 24.0 to reconstruct the surface geometry and divided into sections [34]. Materialize 3‐matic 16.0 software was used to create a 3D model of the femur proximal tip, considering the basic geometrical properties [35]. The subsequently generated model was transferred to the ANSYS Workbench package program for FE analysis (Figure 1) [36].

**FIGURE 2:**
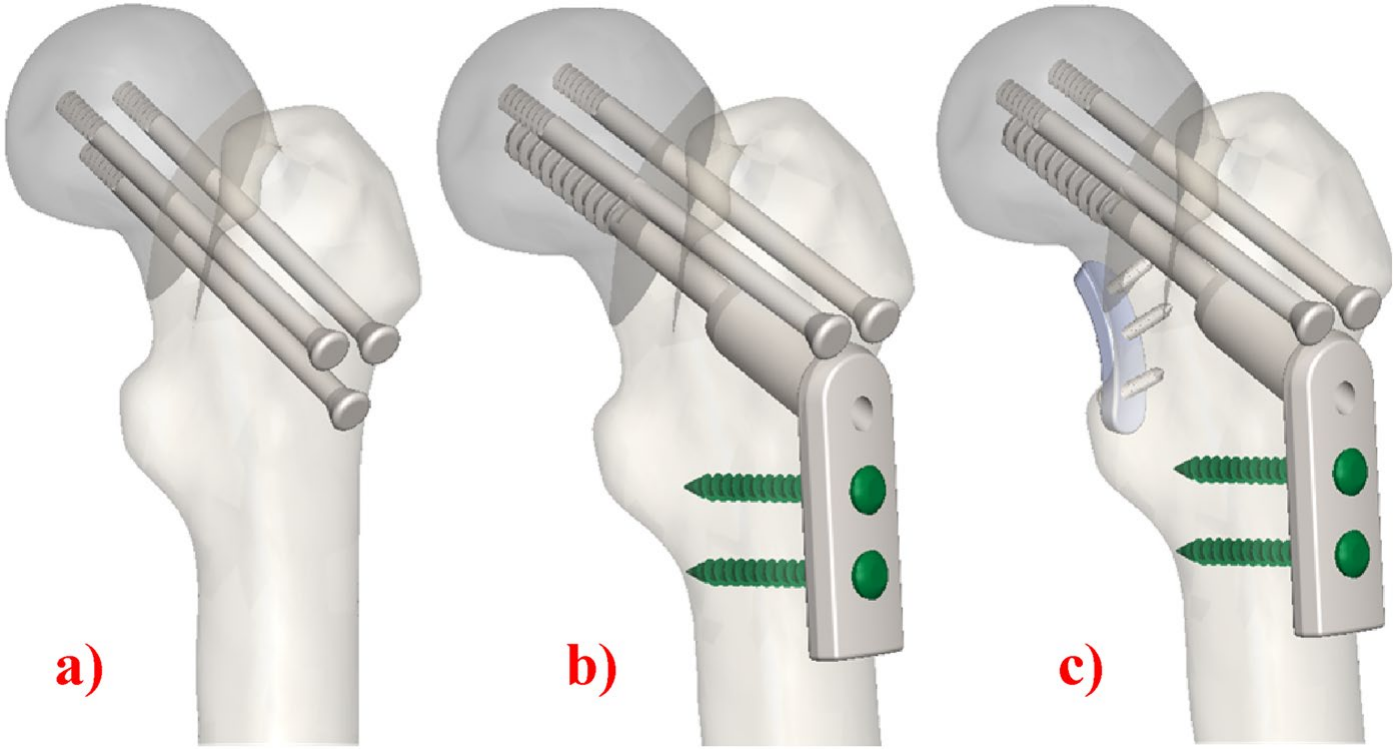
Geometric modeling of internal fixation of FNF.

Three basic fixation methods were used in this study: In the first case, three CS were implanted (Figure 2a); in the second case, the screws were fixed by inserting a plate outside (Figure 2b); in the third case, the screws were implanted by inserting a plate both inside and out (Figure 2c). MBP was placed medially to the femoral neck according to literature [37, 38]. The screws, the geometrical features of which are given in Table 1, were placed perpendicular to the proximal femoral fracture axis and parallel to the three‐dimensional axis of the femoral neck.

**TABLE 1:** Geometric properties of screws and plates (mm).

	Length	Diameter	Thread length
Screw 1	80	7.3	32
Screw 2	85	7.3	32
DHS	80	12.5	22

### 2.2 Material Properties and Mesh Structure

Femoral cortical and cancellous bones are modeled as linear elastic materialsmodels (Table 2). All models in this analysis are taken to be continuous, isotropic, and homogenous linear elastic materials [39, 40].

**TABLE 2:** Material properties used in modeling.

Component	Elastic modulus (MPa)	Poisson's ratio
Cortical bone	16,800	0.3
Cancellous bone	840	0.2
Titanium alloy	105,000	0.35

**FIGURE 3:**
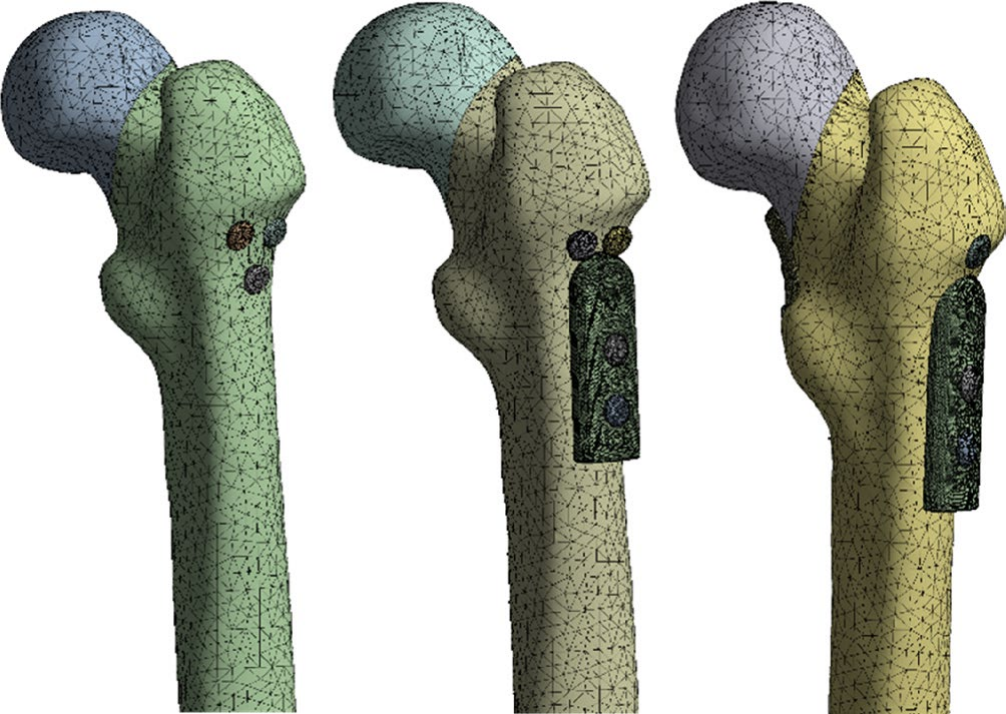
Mesh of the problem.

After the mesh sensitivity analysis, a 3 mm mesh size was chosen for all models. The finite element model consists of a 3D tetrahedron mesh. The three implanted cannular screws were placed parallel to each other. 583,205 elements and 862,117 nodes were used for the obtaining of the FEA results (Figure 3).

### 2.3 Loading and Boundary Conditions

**FIGURE 4:**
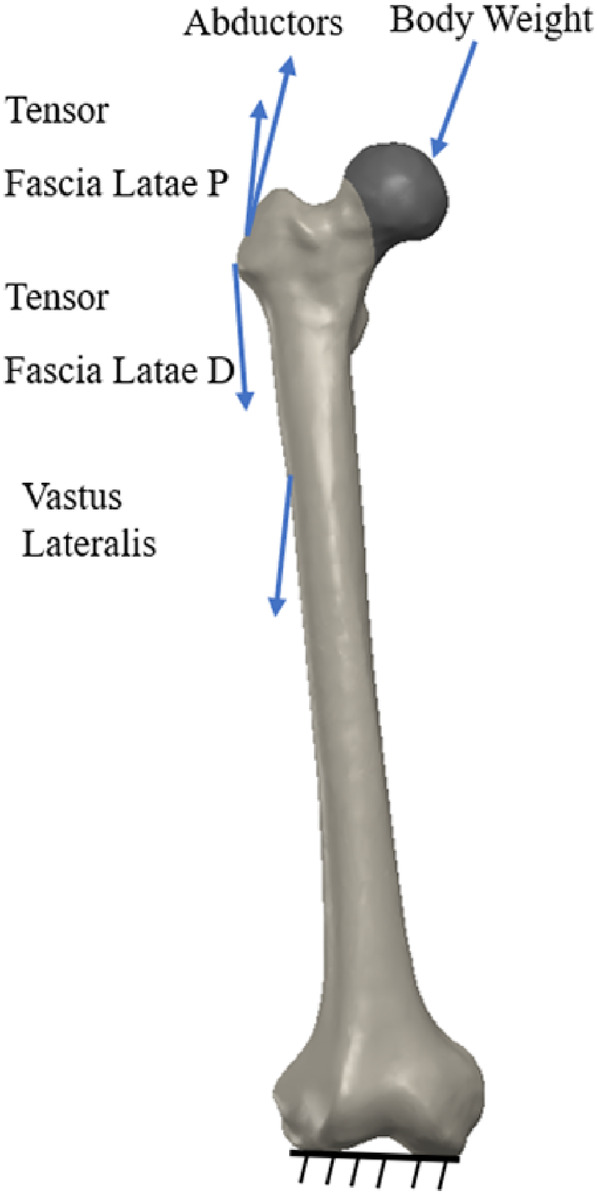
Boundary and loading conditions.

The boundary conditions of the distal femur were restrained, and the degree of freedom in all directions was zero [41]. Figure 4 shows the physical structure of the loading and Table 3 shows its values [41].

**TABLE 3:** Loading values.

Force (*N*)	*F*_*x*_	*F*_*y*_	*F*_*z*_	*F*_total_
Hip joint	378	230	1603	1669.2
Abductors	−406	729.7	−406	729.7
Vastus leteralis	−6.3	663.1	6.3	663.1
Tensor fascialatae lateral part	−3.5	133.2	3.5	133.2
Tensor fascialatae proksimal part	−50.4	−81.2	−92.4	132.9

### 2.4 Contact Settings

According to the sanctioned test contact parameters reported in the literature, binding contact was established between the internal fixation screw and the femur. Friction contact was used on the fracture surface with a friction coefficient 0.46 [43, 44].

### 2.5 Solution

**FIGURE 5:**
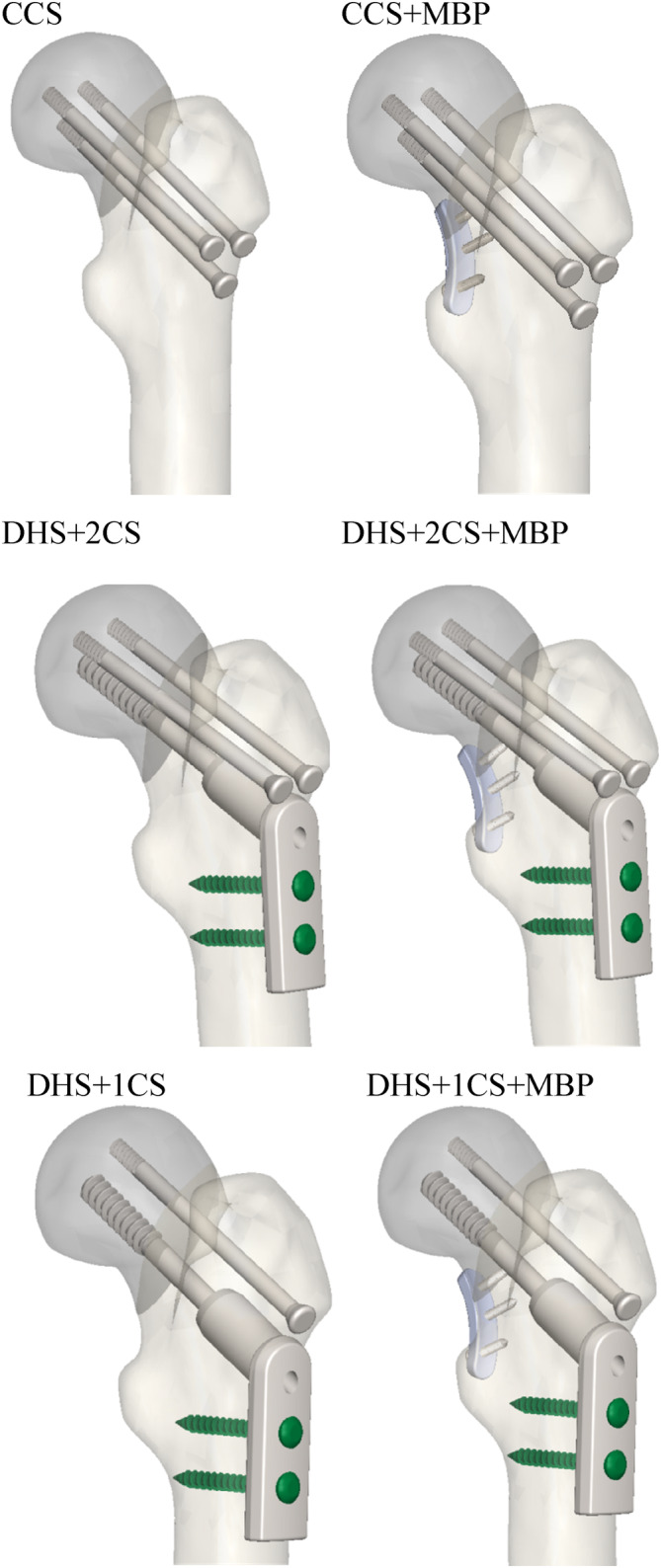
Models of the problem. DHS, dynamic hip screw; CCS, cannulated screw; MBP, medial buttress plate; CCS, CCS+MBP; DHS+2CS, DHS+2CS+MBP; DHS+1CS; DHS+1CS+MBP.

In this study, different treatment practices of proximal femur fracture were observed. In FEA, the crack distance and maximum strain were measured in each group (Figure 5).

## 3 Results

**FIGURE 6:**
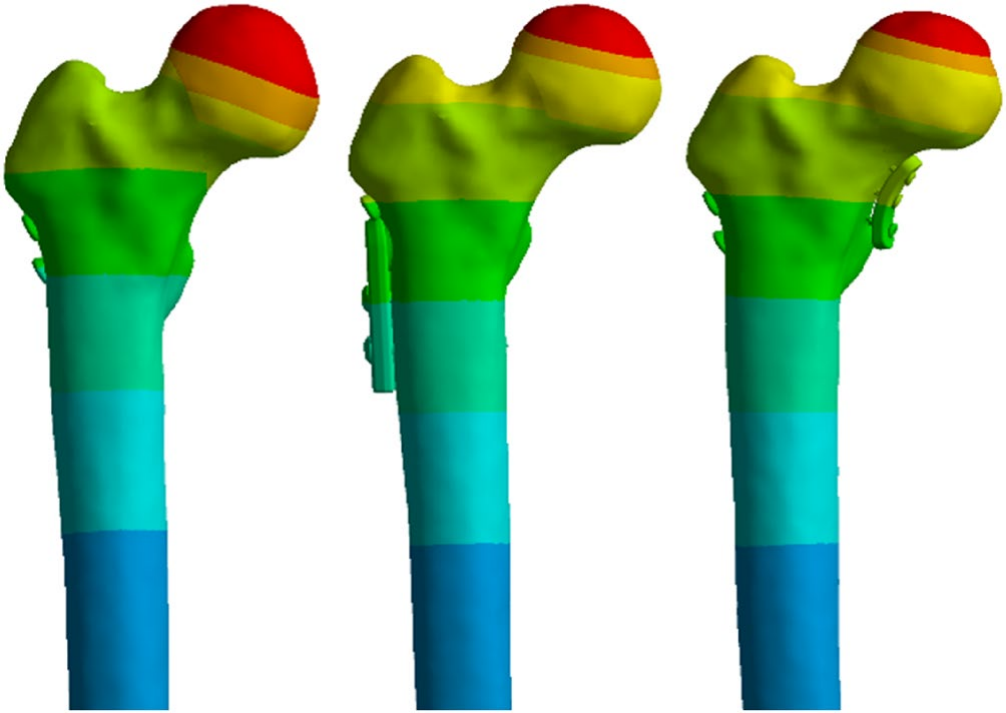
Figures after analysis. Intact‐80: Stiffscrew 80 mm; intact‐85: Stiffscrew 85 mm; LSR: Lower screw release; USR: Upper screw release.

**FIGURE 7:**
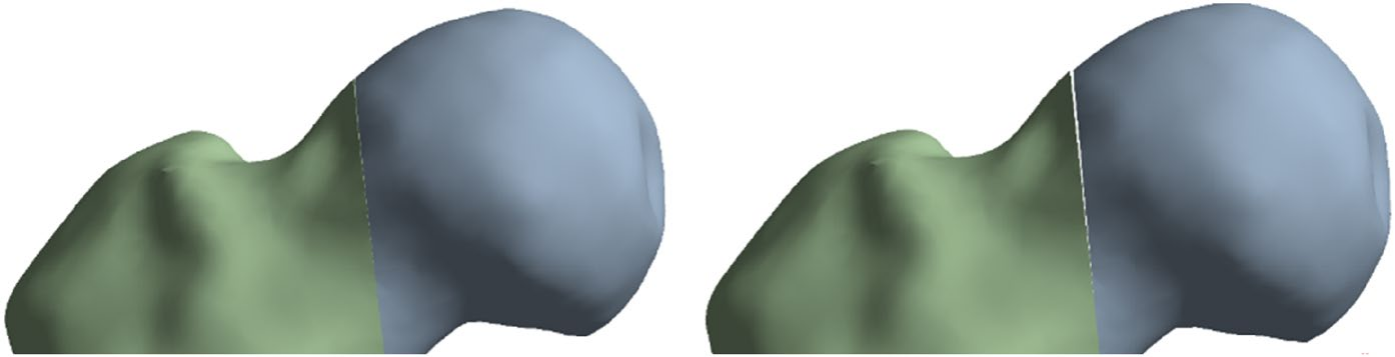
Crack distance.

This study investigated the effects of using different fixation types on crack distances and maximum strain values in the femur using the FEM (Figures 6 and 7). The crack distances and maximum strain values obtained due to the application of different fixation types in different models of the femur are given in Tables 1, 2, 3. Furthermore, the crack images and the distribution of the maximum strain values obtained from the analysis results are given in Figures 1, 2, 3, 4. Parametric expressions in groups were defined below.

Different combinations were considered, as indicated in the tables. In the first case, three CCS were tightened tightly, and the crack distance and maximum strain value were analyzed. Then, the crack distance and the maximum strain values in cases of loosening one screw from the lower, loosening one screw from the upper, loosening the top and lower screws, loosening two screws from the upper, and finally loosening three screws were analyzed.

### 3.1 CCS And CCS+MBP Fixation Types

Analyzing the data presented in Table 4, it is evident that the LSR+USR‐2 model exhibits the greatest crack distance and maximum strain for both CCS and CCS+MBP fixation types. Conversely, the Intact‐85 model displays the lowest crack distance and maximum strain values.

**TABLE 4:** The crack distance and maximum strain for CCS and CCS+MBP fixation types.

Models	Crack distance (mm)		Maximum strain (10^−2^)	
	CCS	CCS+MBP	CCS	CCS+MBP
Intact‐80	0.720	0.680	2.23	2.10
Intact‐85	0.600	0.560	2.18	1.96
LSR	0.800	0.760	2.57	2.43
USR‐1	0.885	0.800	3.20	2.94
LSR+USR‐1	0.900	0.830	3.80	3.72
USR‐2	0.920	0.860	3.85	3.75
LSR+USR‐2	0.982	0.910	4.10	3.95

### 3.2 DHS+2CS and DHS+2CS+MBP Fixation Types

Upon analyzing the data presented in Table 5, it is evident that the LSR+USR‐2 model exhibits the greatest crack distance and maximum strain for both DHS+2CS and DHS+2CS+MBP fixation types. Conversely, the Intact‐85 model displays the lowest crack distance and maximum strain values.

**TABLE 5:** The crack distance and maximum strain for DHS+2CS and DHS+2CS+MBP fixation types.

Models	Crack distance (mm)		Maximum strain (10^−2^)	
	DHS+2CS	DHS+2CS+MBP	DHS+2CS	DHS+2CS+MBP
Intact‐80	0.830	0.780	3.15	2.28
Intact‐85	0.760	0.650	3.05	2.23
LSR	0.900	0.860	3.28	2.90
USR‐1	0.970	0.900	3.35	3.28
LSR+USR‐1	1.000	0.920	3.90	3.85
USR‐2	1.090	0.940	4.05	3.95
LSR+USR‐2	1.200	1.000	4.30	4.20

### 3.3 DHS+1CS and DHS+1CS+MBP Fixation Types

Analyzing the data presented in Table 6, it is evident that the LSR+USR‐2 model exhibits the greatest crack distance and maximum strain for both DHS+1CS and DHS+1CS+MBP fixation types. Conversely, the Intact‐85 model displays the lowest crack distance and maximum strain values.

**TABLE 6:** The crack distance and maximum strain for DHS+1CS and DHS+1CS+MBP fixation types.

Models	Crack distance (mm)		Maximum strain (10^−2^)	
	DHS+1CS	DHS+1CS+MBP	DHS+1CS	DHS+1CS+MBP
Intact‐80	0.860	0.820	3.26	3.10
Intact‐85	0.800	0.700	3.10	2.95
LSR	0.920	0.900	3.35	3.25
USR‐1	0.990	0.950	3.85	3.32
LSR+USR‐2	1.230	1.100	4.40	4.25

### 3.4 Comparison of all Fixation Types

Finally, when Tables 4, 5, 6 were examined, the greatest crack distance and maximum strain values for all fixation types were calculated for the LSR+USR‐2 model. On the other hand, the lowest crack distance and maximum strain values were calculated for the Intact‐85 model.

The greatest crack distance and maximum strain values for all models were obtained in the LSR+USR‐2 model when the DHS+1CS fixation type was used, while the lowest crack distance and maximum strain values were obtained when the CCS+MBP fixation type was used in the Intact‐85 model.

Crack distance and maximum strain values of Intact‐80, Intact‐85, and LSR+USR‐2 models increase for CCS+MBP, CCS, DHS+2CS+MBP, DHS+1CS+MBP, DHS+2CS, DHS+1CS fixation types, respectively.

Maximum strain values for LSR and USR‐1 models increase for CCS+MBP, CCS, DHS+2CS+MBP, DHS+1CS+MBP, DHS+2CS, and DHS+1CS fixation types, respectively. The crack distance values for the LSR and USR‐1 models increase similarly to the maximum strain values. However, equal values were calculated for the DHS+2CS and DHS+1CS+MBP fixation types for the LSR model.

Crack distance and maximum strain values for LSR+USR‐1 and USR‐2 models increase for CCS+MBP, CCS, DHS+2CS+MBP, and DHS+2CS fixing types, respectively. The fixing type values of DHS+1CS and DHS+1CS+MBP were not calculated for these models.

## 4 Discussion

Complications such as shortening, avascular necrosis, femoral varus, and non‐union are more common after treatment of FNF than other fractures. Despite the advancements in treatment methods, effective solutions for these complications have yet to be found. This study aims to examine the possible results and solution suggestions of screw replacement due to various intraoperative reasons in the surgical treatment of FNF from a biomechanical perspective. The study investigated the effect of extending cannulated and lag screws by 0.5 cm on the fracture line following their withdrawal from the treatment. The models were supported with a MBP in high‐stress and deformation cases. When the results were examined, the lowest deformation value was seen in the CCS model. When LSR+USR‐2 models were supported with a MBP, there was a significant decrease in deformation values.

### 4.1 Discussion of CCS and CCS+MBP Fixation Types

**FIGURE 8:**
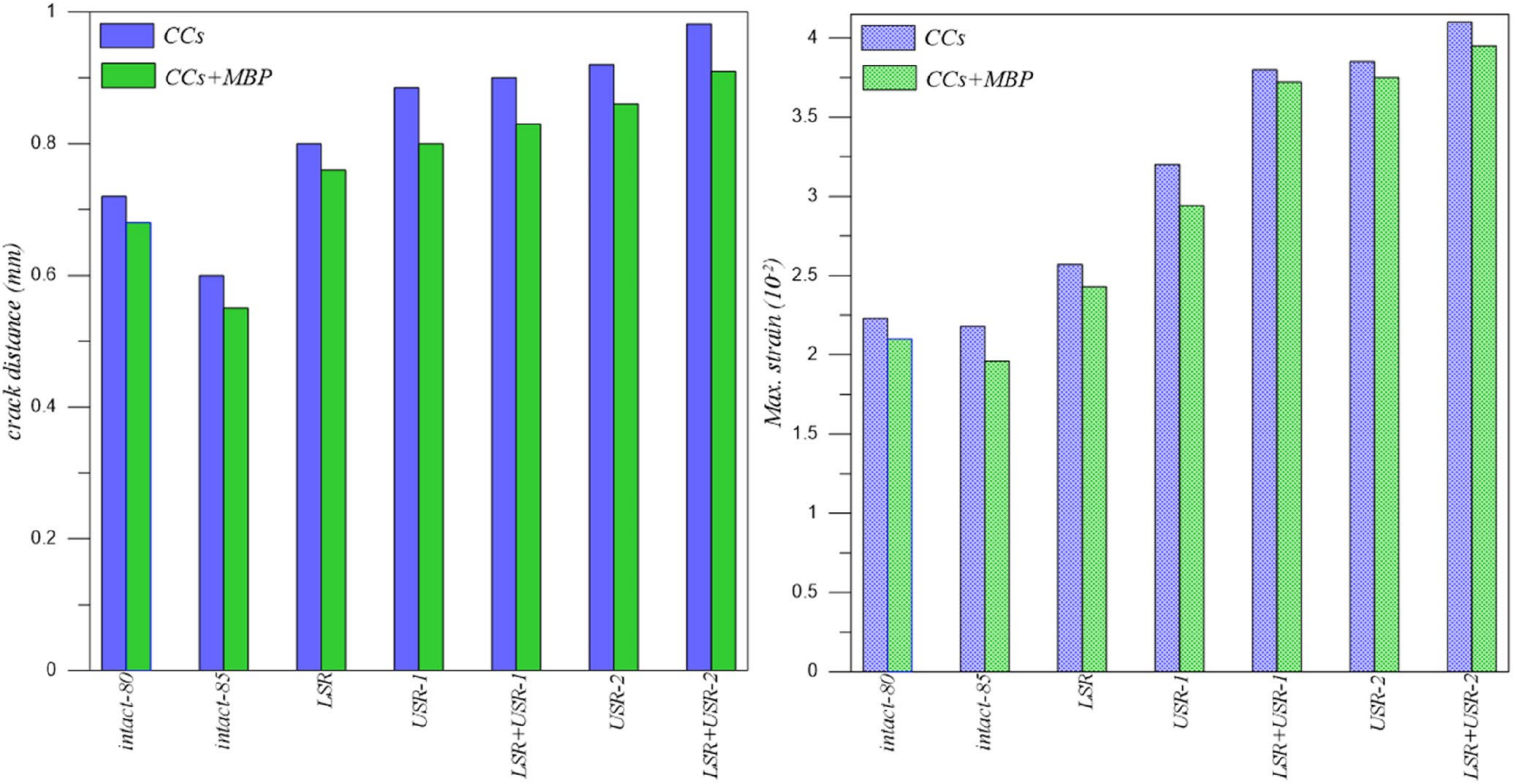
The crack distance and max. strain for CCS and CCS+MBP fixation types.

There is a severe complication rate after the detection of FNF. The large‐series meta‐analysis observed 14.3% avascular necrosis, 9.3% non‐union, 7.1% mal‐union, 9.7% implant failure, and 5.1% surgical site infection [45]. In treating FNF, surgeons use CCS in different configurations and numbers. The standard fixation method usually involves using three partially threaded screws in the form of reverse parallel and reverse triangle. Complication rates due to using CCS are parallel to the general fixation rates. Erivan et al. [46] conducted a study with 112 patients and found that 8.9% of those treated with FNF using CCS had osteonecrosis, while 11.6% experienced non‐union and shortening. To decrease complications, researchers have conducted clinical and biomechanical studies and investigated parameters such as screw configuration, location, size, length, and number. For example, Lim et al. [47] found no association between screw parallelism and fracture non‐union or osteonecrosis. This study observed that using CCS of appropriate size and length provided sufficient compression at the fracture line. Movements in the screws seriously affect the stability of the fracture line. In all models where the CCS was used with the MBP, there was an improvement in the stability of the fracture line, as expected. It can be stated that replacing any of the CCS in the treatment of FNF will not be as stable as not replacing the screws, even if supported by a MBP, as shown in Figure 8.

### 4.2 Discussion of DHS+1CS, DHS+1CS+MBP, DHS+2CS, and DHS+2CS+MBP Fixation Types

**FIGURE 9:**
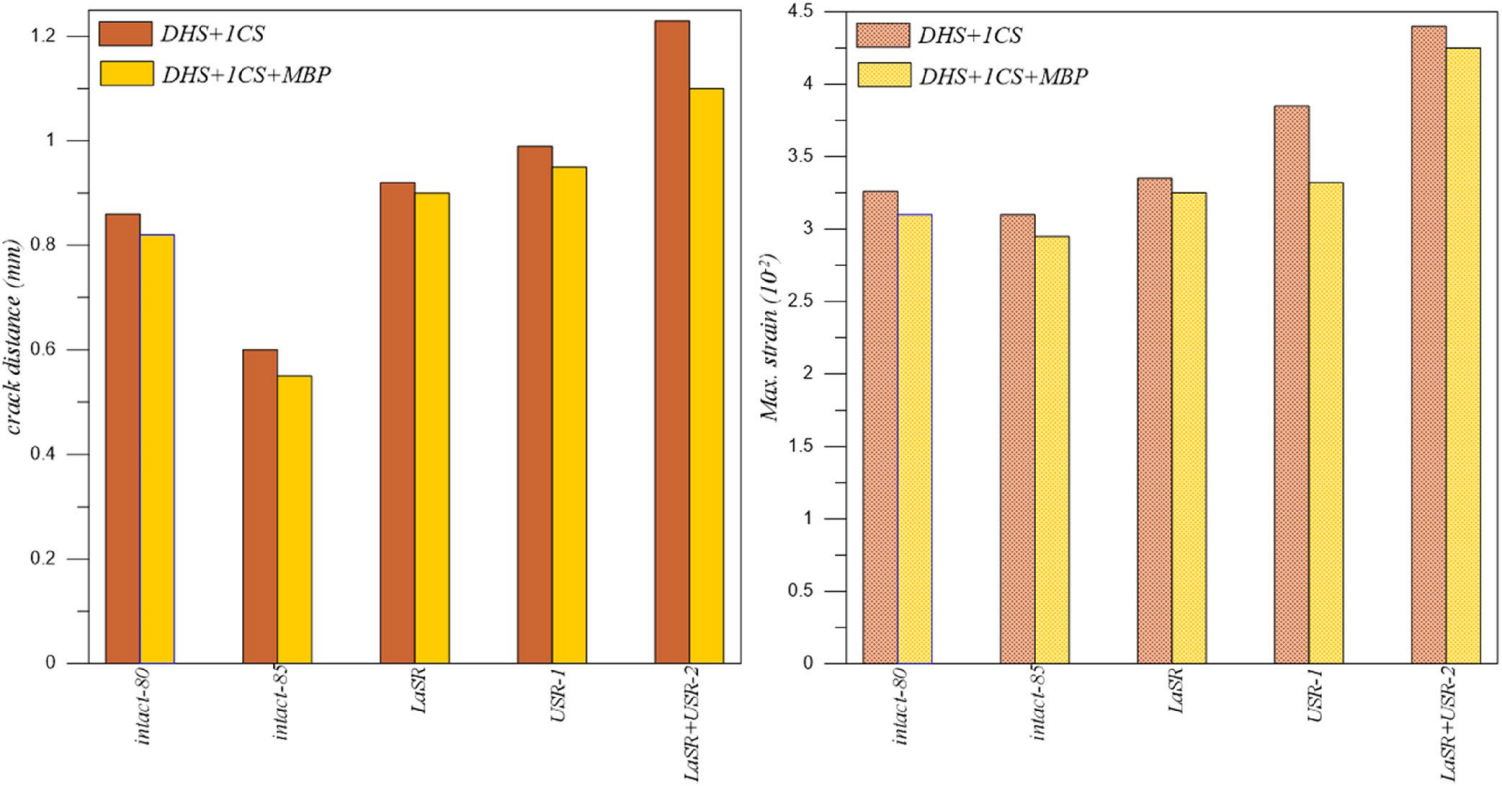
The crack distance for DHS+1CS and DHS+1CS+MBP fixation types.

**FIGURE 10:**
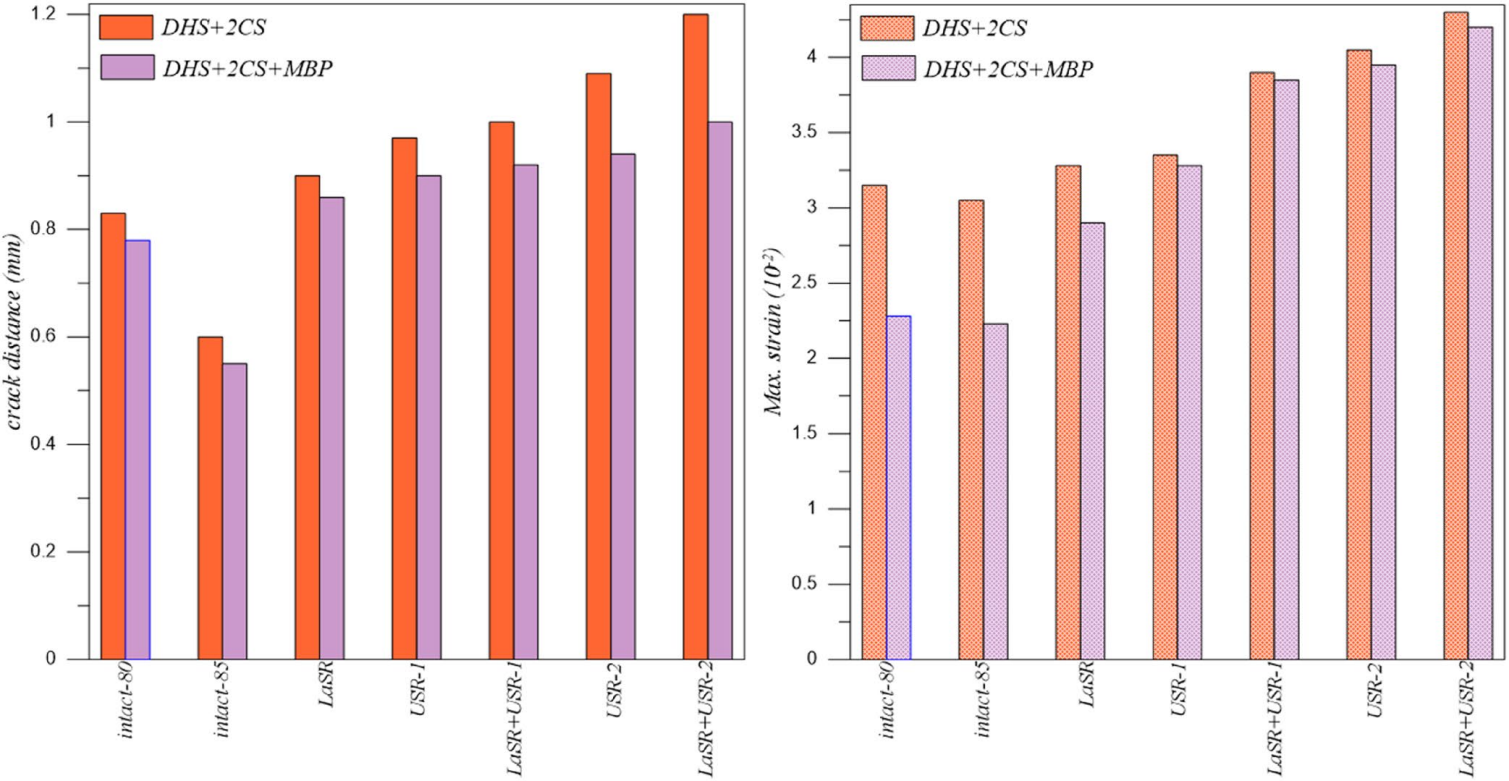
The crack distance for DHS+2CS and DHS+2CS+MBP fixation types.

The use of DHS in treating FNF has been increasing in recent years. Especially with the addition of an anti‐rotation screw, satisfactory results can be achieved in both displaced and non‐displaced fractures [48]. Many meta‐analysis studies are comparing DHS with CCS. Unlike Xia et al. [49] reported a high avascular necrosis rate in DHS and a high implant removal rate in compression screws. They did not report any significant difference in other complications. In this study, DHS generally provide less stability than CCS. If the sliding hip screw is analyzed in isolation, when two anti‐rotation screws are used, its fracture opening and strain value are so low that they cannot take into account. Fracture stability was increased in all models, especially with the USR‐1 model, when an MBP was used (Figures 9 and 10).

### 4.3 Effect on MBP Fixation Types

MBP is mostly used to increase the effect of other fixation methods. In their extensive publications, Collinge et al. [11] investigated the effect of MBP in treating displaced FNF and published interesting results. The authors reported a high rate of complications developed even in surgeries performed by experienced surgeons. However, they encountered a significant decrease in the failure rate in patients using buttress plates. A biomechanical study compared the MBP with the anterior buttress plate. The study found that anterior plate fixation was superior regarding torsional strength and stiffness in a vertical FNF model that used three CCS. The authors reported that the most resistant situations to axial compression were using CCS alone or CCS with MBP. In this study, using a MBP increased fracture stability in all models, as expected.

### 4.4 Strengths and Limitations

Although the FEM used in the field of biomechanics simulates accurate analyses, it will not fully provide the real conditions in some cases. Such studies can be used as a guide for later clinical studies. A notable strength of this study was the lack of a prior biomechanical study evaluating the intraoperative screw loosening scenarios of the most commonly used systems for Pauwels type III fractures.

This study has shown biomechanically that in case of any screw replacement during surgery, the use of MBP will reduce the mechanical values. In addition, in cases of properly placed CCS, it still gives the best values without the need for MBP use. Using number 1 or 2 antirotation screws with DHS does not create major differences in numerical values and it is seen that it provides less compression than CCS screws. MBP can be added to this system in case of any screw loosening during surgery.

## 5 Conclusions

This study includes the possible consequences and solution suggestions of screw replacement for various reasons during FNF surgery. In the study, various implants were placed in the Pauwel type III fracture model with various modifications, and results were obtained under the required loading. Contributions were made to solutions for problems discussed in the literature, such as fracture non‐union and neck shortening.

According to the study results:

- The best results were obtained by using the CCS with the MBP. However, using CCS in the appropriate position and length applicate at once is sufficient. Screw movement requires a MBP for stabilization.
- The DHS plate provides sufficient compression when applied at once and positioned appropriately. However, it cannot provide as much stability as a CCS.
- It is not suitable to support the loosening of the lag screw in the DHS plate with the MBP. The anti‐rotation screw probably contributes to the necessary compression.
- It is unnecessary to support the DHS plate with double anti‐rotation screws.
- The results of this study and literature data show that the method that will provide optimal fracture union and prevent complications has not been fully found. In addition to mechanical studies, intraosseous vascular damage in FNF can be investigated.

## Author Contributions

**Yılmaz Güvercin:** conceptualization, methodology, investigation, writing – original draft, resources. **Murat Yaylacı:** visualization, supervision, methodology, formal analysis, investigation, writing – original draft. **Ayberk Dizdar:** formal analysis, validation, software. **Mehmet Emin Özdemir:** writing – original draft, review and editing. **Sevil Ay:** writing – original draft. **Ecren Uzun Yaylacı:** writing – original draft, review and editing. **Umitcan Karahasanoğlu:** review and editing. **Hüseyin Uygun:** review and editing. **Gökhan Peker:** review and editing.

## Disclosure

All authors listed meet the authorship criteria according to the latest guidelines of the International Committee of Medical Journal Editors, and that all authors are in agreement with the manuscript.

## Ethics Statement

The authors declare that the research was conducted according to the ethical standards.

## Consent

The authors have nothing to report.

## Conflicts of Interest

The authors declare no conflicts of interest.
